# FDG-PET/CT of sarcoidosis and sarcoid reactions following antineoplastic treatment

**DOI:** 10.1186/2193-1801-2-113

**Published:** 2013-03-15

**Authors:** Kentaro Inoue, Ryoi Goto, Hideo Shimomura, Hiroshi Fukuda

**Affiliations:** Department of Nuclear Medicine and Radiology, Institute of Development, Aging and Cancer, Tohoku University, 4-1 Seiryomachi, Aoba-ward, Sendai, 980-8575 Japan

**Keywords:** FDG, PET, Sarcoid reactions, Sarcoidosis, Malignancy, Cancer, Lymphadenopathy

## Abstract

Sarcoidosis or sarcoid reactions, which appear as FDG-avid lesions in oncologic patients, need to be differentiated from disseminated malignancies. We aimed to promote awareness of development of sarcoidosis or sarcoid reactions after antineoplastic therapy to avoid diagnostic errors with FDG-PET/CT findings and assess the utility of FDG-PET/CT for follow-up. We retrospectively reviewed radiological reports of FDG-PET/CT scans performed between January 2009 and December 2011. Among oncologic patients with more than 2 FDG-PET/CT scans, those with nearly symmetrical increases in FDG uptake in the hilar or mediastinal lymph nodes were identified, and those with known sarcoidosis, concurrent diagnoses of sarcoidosis with malignancy, or histopathologically proven malignancies were excluded. Then, only those cases were selected for which sarcoidosis or sarcoid reactions were diagnosed. Four of 376 oncologic cases met the criteria. At 9 months to 6 years after antineoplastic therapy, abnormal FDG uptakes were observed in the hilar, mediastinal, abdominal, pelvic, and inguinal nodes, and/or spleen and lung parenchyma with SUVmax up to 17.7. On the basis of these findings, 1 patient received anticancer chemotherapy because of tumor recurrence suspicion. A gradual decrease in FDG uptake was observed on subsequent PET/CT scans. Sarcoidosis or sarcoid reactions should be considered in differential diagnosis of oncologic patients who have developed FDG-avid lesions any time after antineoplastic therapy. FDG-PET/CT can be used for follow-up in nondiagnostic situations to detect decreases in FDG uptake due to presence of sarcoidal granulomas.

## Introduction

Sarcoidosis is a multisystem granulomatous disease of unknown origin that is characterized by the presence of noncaseating granulomas in affected tissues. Sarcoidal granulomas can involve any organ, but in most cases, intrathoracic lymph-node swelling, pulmonary involvement, and ocular or skin involvement are observed (Iannuzzi et al., 2007). Although the relationship between sarcoidosis and malignancies is controversial, hematological malignancies and solid tumors develop in sarcoidosis patients and sarcoidosis develops in oncologic patients (Cohen and Kurzrock, 2007; Pavic et al., 2008). In oncologic patients, sarcoid reactions (or sarcoid-like reactions), which refer to the development of noncaseating granulomas in patients who do not fulfill the criteria for systemic sarcoidosis, have been observed. Sarcoid reactions occur most commonly in the lymph nodes draining a malignant tumor, but they have also been observed in the stroma, the organ of tumor origin, and distant tissues (Brincker, 1986). The development of sarcoidosis or sarcoid reactions in oncologic patients has been observed after antineoplastic treatment with biologic modifiers such as interferon and interleukin-2, with single or combinations of chemotherapy agents (Cohen and Kurzrock, 2007; Pavic et al., 2008), or even after surgery without chemotherapy (Kennedy et al., 2008).

Positron emission tomography (PET) and PET/computed tomography (CT) with 2-deoxy-2-[F-18]fluoro-D-glucose (FDG) have been widely used in the management of malignancy based on the increased use of glucose by malignant cells. The use of FDG-PET/CT for staging, assessing the response to therapy, and diagnosing the recurrence of malignancy is, however, hampered by active inflammation and granulation tissues, which are also known to accumulate FDG (Shreve et al., 1999). Active sarcoidosis shows high FDG uptake and, therefore, can be a pitfall for the diagnosis of malignancies in one respect, but repeated FDG-PET can be used for the assessment of the activity of sarcoidosis and its response to therapy (Teirstein et al., 2007; Braun et al., 2008).

With the increasing use of FDG-PET/CT, a number of studies have described that the development of sarcoidosis or sarcoid reactions results in FDG uptake that needs to be differentiated from disseminated malignancies in patients with a history of treatment for malignancies, within a few months to more than 10 years after the diagnosis of the malignancy (Chowdhury et al., 2009; Hunt et al., 2009).

We aimed to promote awareness about the development of sarcoidosis or sarcoid reactions after antineoplastic therapy in order to avoid diagnostic errors with FDG-PET/CT findings and assess the utility of FDG-PET/CT for follow-up in this situation. Therefore, we describe FDG-PET/CT findings and the time course around the detection of FDG-avid lesions in patients with sarcoidosis or sarcoid reactions that developed after antineoplastic therapy.

## Materials and methods

We retrospectively reviewed the radiological reports of PET/CT scans that were performed at our university hospital between January 2009 and December 2011. Among the patients who underwent oncologic FDG PET/CT scans, those who had undergone more than 2 FDG PET/CT scans were selected. Patients with known sarcoidosis, a concurrent diagnosis of sarcoidosis, or a histologically proven recurrence of a malignancy, were excluded. Patients who were referred from other hospitals to our department only for PET/CT scans were also excluded due to lack of other clinical data. By analyzing radiological reports, patients with abnormal increases in FDG uptake in the hilar and/or mediastinal lymph nodes during a series of PET/CT scans were identified as cases of suspected sarcoidosis that developed or flared after antineoplastic therapy. Then, further information was obtained from the clinical and pathological data, and patients diagnosed with sarcoidosis or sarcoid reactions were selected. Imaging and clinical data from before 2009 were obtained for those patients if available. Informed consent was waived for this retrospective study. The protocols of this study were approved by the Ethical Committee of the Tohoku University Graduate School of Medicine.

All integrated PET/CT images were obtained at our institution with a Biograph Duo or a Biograph 40 PET/CT system (Siemens AG, Erlangen, Germany) using our standard clinical protocol. The patients fasted for at least 4 h. An intravenous injection of 185–370 MBq (5-10 mCi) of FDG was administered. Then, the patients rested in a reclining chair in a quiet room for 1 h between the tracer injection and the start of PET/CT image acquisition. No oral or intravenous contrast agent was used. FDG-PET emission data were typically obtained for 2 min in each bed position during the emission scan. Patients were instructed to continue shallow breathing during PET acquisition. FDG-PET images were reconstructed using the CT transmission map for attenuation correction. FDG-PET/CT images were interpreted in standard clinical fashion by 2 nuclear radiologists.

## Results

Of the 376 oncologic patients who underwent more than 2 FDG-PET/CT scans during follow-up after antineoplastic therapy, 4 patients were diagnosed with sarcoidosis or sarcoid reactions based on histopathological and clinical data. There were several patients in whom sarcoidosis or sarcoid reactions could be suspected based on the PET/CT findings, but the diagnosis was not confirmed clinically or histopathologically. These unconfirmed cases were not included in this study because the aim of this study was not to indicate the prevalence of sarcoidosis or sarcoid reactions in patients who underwent oncologic FDG-PET/CT scans after antineoplastic treatment. The primary malignancy was carcinoma in 3 patients and osteosarcoma in 1 patient. The detailed clinical histories of the patients are described as follows.

### Case 1

**Figure 1:**
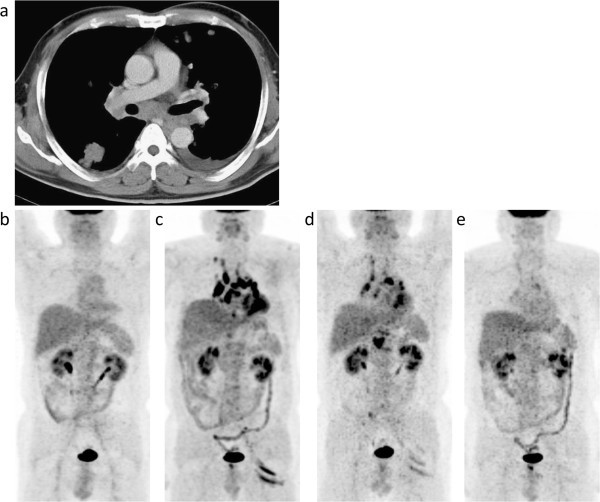
**Case 1.** A contrast-enhanced CT image taken at the time of the diagnosis of nonseminomatous malignant germ cell tumors with multiple intrathoracic and abdominal lymph nodes and lung metastases **(a)** and maximum intensity projection (MIP) images of FDG-PET/CT scans that were conducted 3 **(b)** and 6 years **(c)** after the completion of antineoplastic therapy and 8 **(d)** and 15 months **(e)** after the detection of FDG-avid lesions. **(a)** CT images before antineoplastic therapy showing metastases in the bilateral hilar and mediastinal nodes and lungs with pleural effusion. **(b)** No abnormal FDG uptake is observed. **(c)** Increased FDG uptake is evident in the bilateral hilar and mediastinal nodes (maximum standardized uptake value (SUVmax) 17.7), abdominal nodes, and the left gluteal muscles. **(d, e)** A gradual decrease to normal is observed concerning FDG uptake in these lesions.

A 43-year-old man was treated for nonseminomatous malignant germ cell tumors with multiple intrathoracic and abdominal lymph nodes and lung metastases (Figure 1a) by chemotherapy (1 cycle of the combination of etoposide and cisplatin, 2 cycles of the combination of bleomicyn, etoposide and cisplatin, and, then, 2 cycles of the combination of etoposide, carboplatin and ifosfamide), followed by the removal of the residual retroperitoneal tumor and neck lymph nodes. After the completion of therapy, lymph node swelling and lung metastases were diminished. An FDG-PET/CT scan that was performed 3 years after the completion of therapy showed no abnormal findings (Figure 1b). Six years after the completion of the therapy, mediastinal and hilar node swelling was found on a CT scan (not shown). He was therefore referred to our department for an FDG-PET/CT study because recurrence was suspected. Before therapy, the patient’s serum human chorionic gonadotropin and α-fetoprotein levels were 65,800 mIU/mL (normal range < 5 mIU/mL) and 162.2 ng/mL (normal range < 20 ng/mL), respectively, which have remained normal since the completion of therapy.

The FDG-PET/CT scan showed multiple foci of abnormal uptake in the mediastinal and bilateral hilar nodes, abdominal nodes such as the retrocrural and para-aortic nodes, and left iliac artery nodes (Figure 1c). Uptake in the left gluteal muscle was also noted. Because of previous tumor involvement in the intrathoracic nodes, a suspicion of tumor recurrence was reported to the referring physicians. One cycle of chemotherapy was administered. No change in the lymph nodes swelling was noted on a CT scan, and a subsequent FDG-PET/CT scan did not reveal a significant change (not shown). Thus, a suspicion of sarcoidosis or sarcoid reactions was reported, and histological sampling from the mediastinal lymph nodes by endobronchial ultrasound-guided transbronchial needle aspiration confirmed noncaseating granulomas without the evidence of a malignant tumor. Follow-up was arranged thereafter to rule out recurrence. FDG-PET/CT scans that were performed 8 and 15 months after the detection of abnormal FDG uptake revealed gradual decrease in abnormal uptake (Figure 1d and e). During the course of follow-up, the levels of serum angiotensin converting enzyme (ACE) were normal. Tumor recurrence has not been observed, and without evidence of ocular, cardiac, lung, or skin sarcoid granulomas, a diagnosis of sarcoid reactions was made.

### Case 2

**Figure 2:**
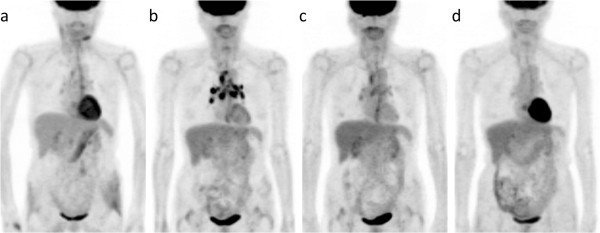
**Case 2.** FDG-PET/CT images that were conducted 0 **(a)** and 9 months **(b)** after the completion of antineoplastic therapy for mandibular osteosarcoma and 6 **(c)** and 28 months **(d)** after the detection of FDG-avid lesions.

A 64-year-old woman underwent segmental resection of the mandible for an osteosarcoma, which was followed by chemotherapy (adriamycin) due to a positive margin. On an FDG-PET/CT scan taken at the end of chemotherapy, only mild and nonspecific FDG uptake was observed in the intrathoracic nodes (Figure 2a). Then, a FDG-PET/CT scan that was performed 9 months after therapy depicted high FDG uptake in the mediastinal and bilateral hilar nodes (Figure 2b). Mediastinal node biopsy revealed noncaseating granulomas with a negative stain for acid-fast bacilli and fungi. Serum ACE levels were assessed due to a suspicion of sarcoidosis, and were found to be marginally elevated (26.1 U/L, normal range 7-25 U/L). Her serum ACE levels decreased to 20.7 U/L two months after the PET/CT scan and remained within a normal range for 2 years. Sarcoidosis was diagnosed on the basis of these findings, and the patient was observed thereafter without treatment for sarcoidosis or malignancy. Follow-up PET/CT scans were performed and they revealed decreases in FDG uptake in the mediastinal and hilar nodes (Figure 2c and d). Neither progression of sarcoidosis nor metastasis of the osteosarcoma has been found.

### Case 3

A 54-year-old woman presented with an oral floor tumor. After being diagnosed with oral floor carcinoma, she was treated with chemoradiotherapy (cisplatin, fluorouracil, docetaxel, and a total of 70 Gy of radiation), and then, due to the growth of the residual tumor, total glossolaryngectomy with bilateral neck dissection was performed.

**Figure 3:**
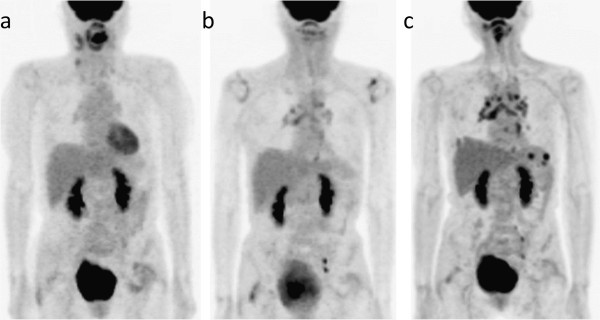
**Case 3.** MIP images of FDG-PET(/CT) scans that were obtained at the time of the diagnosis of the malignancy **(a)** and 9 months **(b)** and 2 years **(c)** after the completion of antineoplastic therapy.

An FDG-PET/CT scan that was conducted before therapy showed high FDG uptake in the primary tumor, as well as in the right neck node metastatic lesions, without significant uptake in the intrathoracic nodes (Figure 3a). Retrospectively, mild FDG uptake was noted in the mediastinal and bilateral hilar nodes on a PET scan that was performed 9 months after therapy (Figure 3b). However, these findings were determined to be due to non-specific reactive uptake. On an FDG-PET/CT scan taken 2 years after therapy for restaging, moderate FDG uptake was noted in the mediastinal and bilateral hilar nodes, spleen, and right lung (Figure 3c). Histological sampling from the mediastinal node by transbronchial lymph node biopsy confirmed the presence of noncaseating granulomas without evidence of malignant tumor cells. Stains and cultures for acid-fast bacilli and fungi were negative. Accordingly, a diagnosis of sarcoidosis was made.

### Case 4

A 54-year-old woman presented with a tumor in her left breast. After being diagnosed with invasive ductal carcinoma, she underwent chemotherapy (cyclophosphamide, epirubicin and fluorouracil) followed by mastectomy and axillary lymph node dissection. At the time of diagnosis, mediastinal and hilar lymphadenopathy was not observed. Follow-up revealed no recurrence for 4 years after therapy, but intrathoracic lymph node swelling was subsequently detected by a CT scan (not shown). She was referred to our department for a FDG-PET/CT scan because recurrence was suspected.

**Figure 4:**
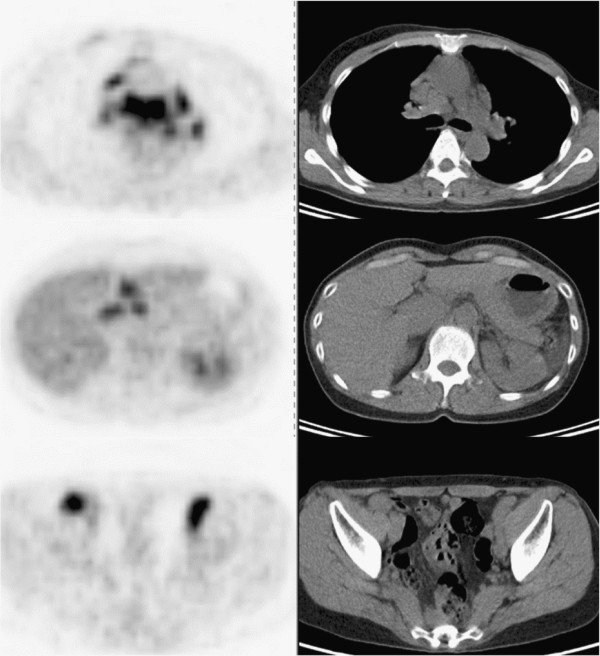
**Case 4.** Axial FDG-PET/CT images obtained 4 years after antineoplastic therapy for left breast cancer (left column, FDG-PET; right column, CT).

On the FDG-PET/CT scan, there were multiple foci of abnormal uptake in the bilateral supraclavicular nodes, multiple mediastinal and bilateral hilar nodes, and abdominal, iliac and bilateral inguinal nodes (Figure 4). Due to the suspicion of malignant lymphoma, sarcoidosis, or metastases, an axillary node was biopsied, and noncaseating granuloma was detected without evidence of tumor recurrence. Stains and cultures for acid-fast bacilli and fungi were negative. In addition, increased levels of serum ACE were noted (53.0 U/L). She was then diagnosed with sarcoidosis. Another FDG-PET/CT scan that was performed 6 months after the diagnosis of sarcoidosis did not show remarkable changes (not shown). Her serum ACE levels decreased to 44.4 U/L 14 months after the diagnosis of sarcoidosis, but they have not returned to normal after 2 years. She has been followed up for sarcoidosis, and no recurrence of cancer has been observed.

## Discussion

We identified 4 patients with sarcoid reaction (n=1) and flare or new onset of sarcoidosis (n=3) who had been treated for malignancy and exhibited FDG-avid lesions on PET/CT scans. Abnormal uptake on FDG-PET/CT was observed in the intrathoracic, abdominal, pelvic and inguinal nodes and/or spleen and lung parenchyma, with maximum standardized uptake value up to 17.7, 9 months to 6 years after antineoplastic therapy.

The present patients had different malignant tumors and received different antineoplastic therapies; all patients received chemotherapy, albeit with different regimens, with or without surgery and radiotherapy. As observed in the present study, the new onset of previously undiagnosed sarcoidosis or the development of sarcoid reactions has been observed in patients with diverse malignant neoplasms. This observation was more common in patients with hematological malignancies, such as malignant lymphoma (Suen et al., 1990; Hunsaker et al., 1996; Goswami et al., 2010), but it was also observed in patients with solid tumors, such as testicular cancer (Tjan-Heijnen et al., 1998; Karapetis et al., 2001), breast cancer (Hunsaker et al., 1996; Hunt et al., 2009), lung cancer (Maeda et al., 2005; Chida et al., 2010; Umezu et al., 2010), head and neck cancer (Yao et al., 2005), osteosarcoma (Okada et al., 2005) and cancers of the digestive tract and genitourinary system (Cohen and Kurzrock, 2007; Pavic et al., 2008; Chowdhury et al., 2009). Antineoplastic treatment-associated sarcoidosis has been commonly linked with interferon-α and γ (Cohen and Kurzrock, 2007; Pavic et al., 2008). The onset of sarcoidosis has, however, been preceded by several other agents and combination chemotherapy (Cohen and Kurzrock, 2007), as observed in the present cases, and in cases that did not involve antineoplastic chemotherapy (Suen et al., 1990; Hunt et al., 2009). The type of preceding malignancy or antineoplastic therapy, as well as the duration after the completion of therapy is, therefore, not sufficient for distinguish a disseminated malignancy from sarcoidosis or sarcoid reactions after antineoplastic therapy.

The relationship between malignancy and subsequent sarcoidosis, as well as that between sarcoidosis and subsequent malignancy, has been controversial (Cohen and Kurzrock, 2007). In line with the notion that sarcoidosis results from the exposure of genetically susceptible individuals to specific agents (American Thoracic Society, 1999), antineoplastic therapy may lead to the release of antigens that can trigger the onset of sarcoidosis; however, the association might be fortuitous in some cases (Brincker, 1995; Reich et al., 1995). Tumor-related sarcoid reactions have also been considered to be caused by soluble antigens that are derived from tumor cells (Brincker, 1986). Sarcoidosis should be diagnosed by excluding the many diseases that are capable of producing similar granulomas as well as tumor-related sarcoid reactions (American Thoracic Society, 1999). However, the distinction between sarcoidosis occurring in an oncologic patient and sarcoid reactions is difficult. The affected tissues of sarcoid reactions are histologically identical to granulomas in sarcoidosis, except that granulomas in sarcoid reactions are B-cell positive whereas those in sarcoidosis are B-cell negative (Brincker and Pedersen, 1991). The radiographical pattern of disease is not distinctive between sarcoidosis and sarcoid reactions (Hunsaker et al., 1996). Sarcoid reactions, however, can occur with concomitant metastasis, although this is less frequent than its occurrence without metastasis (Brincker, 1986). Therefore, a recurrent tumor should be cautiously excluded in cases in which the diagnosis of systemic sarcoidosis cannot be confirmed.

FDG-PET(/CT) is a useful technique both in managing a malignancy and assessing the extent of the organ involvement of sarcoidosis (Teirstein et al., 2007; Braun et al., 2008). Regarding the differential diagnosis of disseminated malignancies from sarcoidosis or sarcoid reactions, symmetrical hilar FDG uptake may be related to a benign cause (Karam et al., 2008). It is however difficult to apply this finding to patients with previous thoracic node involvement of malignancies such as the present Case 1. In addition, the characteristics of hilar FDG uptake in patients with granulomatous disease can be similar to those in patients with metastatic disease (Karam et al., 2008). Additionally, the differential diagnosis for newly developed multiple FDG-avid lesions in lymph nodes, spleen, and lung parenchyma as found in the present study would overlap those of sarcoidosis including malignant lymphoma, infectious diseases such as mycobacteriosis and mycosis, and lymphadenitis (American Thoracic Society, 1999). Therefore, the utility of FDG-PET(/CT) is in pinpointing the organs that are candidates for diagnostic biopsy and not distinguishing between the disseminated malignancy and granulomatous and inflammatory diseases. In addition, any clinical data, such as the levels of tumor markers as in the present Case 1, should be utilized in reporting FDG-PET findings, if available, to suggest a differential diagnosis other than metastatic malignancy for FDG-avid lesions to referring physicians.

In the present study, gradual decreases in FDG uptake were observed during follow-up after the detection of FDG-avid lesions in 2 patients. Nearly two-thirds of patients with sarcoidosis have a spontaneous remission (American Thoracic Society, 1999; Iannuzzi et al., 2007). More importantly, the decrease in FDG uptake could be a marker to rule out recurrent malignancy in oncologic patient who have neither histopathological proof of metastasis of malignancy nor fulfill the criteria for systemic sarcoidosis as in the present Case 1. However, the long duration to confirm a remarkable decrease in FDG uptake is a disadvantage, and even 6 months would not be sufficient for judging relevant changes in FDG uptake, as observed in the present Case 4. To overcome this disadvantage, FDG-PET(/CT) could be used to assess early metabolic responses to corticosteroid therapy to discriminate sarcoidosis and sarcoid reactions from malignancy in some cases (Aide et al., 2009). Conversely, the spontaneous remission of sarcoidosis or sarcoid reactions could mimic the regression of the malignancy if antineoplastic therapy is performed on the basis of a misdiagnosis of malignancy and sarcoidosis. PET using F-18-alpha-methyltyrosine could be used to distinguish sarcoidosis from malignancy, although malignancy would not be excluded if the findings were negative (Kaira et al., 2007).

In the present study, only patients who displayed abnormal increases in FDG uptake in the intrathoracic nodes were selected as possible cases of sarcoidosis or sarcoid reactions on the basis of preceding reports (Hunsaker et al., 1996; Chowdhury et al., 2009; Hunt et al., 2009). In a few reports, however, sarcoid reactions after antineoplastic therapy have been observed only in the spleen (Reilly et al., 2007), or axial lymph nodes (Martella et al., 2012). Therefore, our study may have failed to include cases of sarcoidosis or sarcoid reactions that occurred in organs other than intrathoracic nodes. In future investigations, we should attempt to distinguish sarcoid reactions from malignancy when we observe the development of FDG-avid lesions without the involvement of the intrathoracic nodes.

In conclusion, the present study demonstrated that the occurrence of a sarcoidosis or sarcoid reaction was observed in patients with several types of solid malignant tumors with variable durations since the end of theraphy and with variable types of antineoplastic therapy. FDG-avid lesions due to sarcoidosis or sarcoid reactions could mimic the recurrence of malignancy in oncologic patients. Symmetrical FDG uptake in the hilar and/or mediastinal nodes is a typical finding of sarcoidosis and sarcoid reactions on FDG-PET(/CT). Therefore, these conditions should not be discounted even in patients with malignancies that previously involved the intrathoracic nodes.
